# Eight Questions About Physician-Rating Websites: A Systematic Review

**DOI:** 10.2196/jmir.2360

**Published:** 2013-02-01

**Authors:** Martin Emmert, Uwe Sander, Frank Pisch

**Affiliations:** ^1^Institute of Management (IFM)School of Business and EconomicsFriedrich-Alexander-University Erlangen-NurembergNurembergGermany; ^2^University of Applied Sciences and ArtsHannoverGermany

**Keywords:** Physician rating websites, patient narratives, public reporting, transparency, systematic review

## Abstract

**Background:**

Physician-rating websites are currently gaining in popularity because they increase transparency in the health care system. However, research on the characteristics and content of these portals remains limited.

**Objective:**

To identify and synthesize published evidence in peer-reviewed journals regarding frequently discussed issues about physician-rating websites.

**Methods:**

Peer-reviewed English and German language literature was searched in seven databases (Medline (via PubMed), the Cochrane Library, Business Source Complete, ABI/Inform Complete, PsycInfo, Scopus, and ISI web of knowledge) without any time constraints. Additionally, reference lists of included studies were screened to assure completeness. The following eight previously defined questions were addressed: 1) What percentage of physicians has been rated? 2) What is the average number of ratings on physician-rating websites? 3) Are there any differences among rated physicians related to socioeconomic status? 4) Are ratings more likely to be positive or negative? 5) What significance do patient narratives have? 6) How should physicians deal with physician-rating websites? 7) What major shortcomings do physician-rating websites have? 8) What recommendations can be made for further improvement of physician-rating websites?

**Results:**

Twenty-four articles published in peer-reviewed journals met our inclusion criteria. Most studies were published by US (n=13) and German (n=8) researchers; however, the focus differed considerably. The current usage of physician-rating websites is still low but is increasing. International data show that 1 out of 6 physicians has been rated, and approximately 90% of all ratings on physician-rating websites were positive. Although often a concern, we could not find any evidence of "doctor-bashing". Physicians should not ignore these websites, but rather, monitor the information available and use it for internal and ex-ternal purpose. Several shortcomings limit the significance of the results published on physician-rating websites; some recommendations to address these limitations are presented.

**Conclusions:**

Although the number of publications is still low, physician-rating websites are gaining more attention in research. But the current condition of physician-rating websites is lacking. This is the case both in the United States and in Germany. Further research is necessary to increase the quality of the websites, especially from the patients’ perspective.

## Introduction

Creating more transparency about the quality of health care providers has become a major challenge in delivering more effective and efficient health care quality [1,2]. According to the theory of Public Reporting (PR), patients are expected to inform themselves about the quality of participants in the health care system (eg, physicians, hospitals, health plans) before making decisions and selecting health care providers [3-5]. The newest development within this movement is physician rating websites, which are gaining popularity among patients [6-8]. It is noteworthy that there are already PR instruments in place, such as the New York State Cardiac Surgery Reporting System (CSRS) (eg, [9-11], Nursing Home Compare [12], or the German Klinikführer Rhein-Ruhr [13]. However, physician-rating websites are a consumer-driven alternative [14]. Traditional PR initiatives generally assess the quality of care of health care providers by measuring adherence to clinical guidelines, and some also include information on patients’ satisfaction [2]. In contrast, the primary focus of physician-rating websites lies in rating and discussing the performance of physicians; however, one can also find addresses, opening hours, and certification of the physicians [2]. Although the usefulness of physician-rating websites has been seen as critical [6], greater importance must be assumed [7,15].

In this paper, we summarize the existing literature on physician-rating websites based on a systematic review of published articles. Our objective was to provide a structured, comprehensive overview of the available evidence on physician-rating websites. Therefore, we addressed the following eight topics: 1) What percentage of physicians has been rated? 2) What is the average number of ratings on physician-rating websites? 3) Are there any differences among rated physicians related to socioeconomic status? 4) Are ratings more likely to be positive or negative? 5) What significance do patient narratives have? 6) How should physicians deal with physician-rating websites? 7) What major shortcomings do physician-rating websites have? 8) What recommendations can be made for further improvement of physician-rating websites?

## Methods

For this review, we adhered to guidelines from the Cochrane Collaboration [16], the Institute for Quality and Efficiency in Health Care [17], the Hannoveraner Konsensus [18], and the NHS Economic Evaluation Database [19]. In total, we searched the following seven databases: Medline (via PubMed), the Cochrane Library, Business Source Complete, ABI/Inform Complete, PsycInfo, Scopus, and ISI web of knowledge. Articles published prior to May 2012 were eligible for inclusion. We also included commentaries, discussion papers, etc., if published in peer-reviewed journals. The focus of the article had to deal with websites on which individual physicians (ie, not entire hospitals) could be rated.

Our search strategy was segmented into two components (the search history is available upon request from the first author). The first component referred to physicians (eg, physicians, doctors, or health care providers), and the second to online rating websites (eg, rating sites, rating websites, review sites, review websites, websites to assess, Internet ratings, online ratings, web ratings, online reviews, opinion websites, experience websites, online physician ratings, online doctor ratings, online provider ratings, or public reporting). Search terms included both singular and plural. Search terms from previously published studies were used (eg, [2,6,14,20-22] and further expanded. To ensure that relevant documents would not be missed, we also searched the Internet via Google, Google Scholar, and reviewed reference lists.

Two authors independently reviewed all papers generated by the search procedure and assessed their eligibility for inclusion (discussion between the 2 authors resolved the few disagreements). They also independently extracted relevant information from identified articles. Both authors used the same abstraction form, containing the following elements: authors, year of publication, country, assessed physician-rating websites, and the information relevant to our questions (see above). Again, discussion between the 2 authors resolved the few minor differences that emerged. Due to heterogeneity of the studies, no study appraisal was carried out. As a minor requirement, we defined publishing in a scientific journal with a peer-review process.

## Results

### Search Results

**Figure 1:**
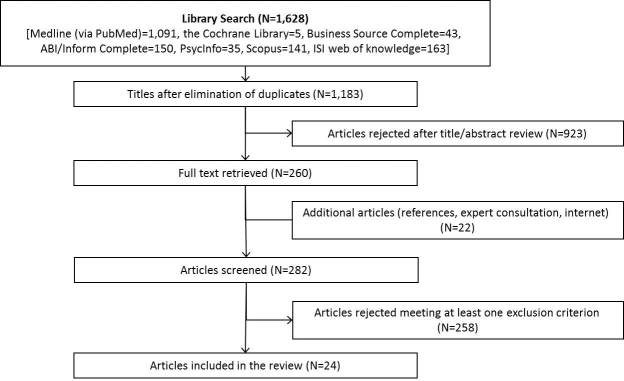
Search results.

The initial search identified 1628 articles. After eliminating duplicates and a review of titles and abstracts, 260 studies remained for detailed reflection (see Figure 1). Screening of reference lists, expert consultation, and Internet searches yielded 22 additional articles. Finally, 24 articles met our inclusion criteria. It is worth mentioning that the papers included vary considerably by inclusion criteria and focus. The result is a wide range in the number of included studies. Furthermore, all studies were published either in English or German. We did not find a study containing an English language abstract in another language (eg, Spanish, French) during our review process. Most papers have been published by US (n=13) or German authors (n=8). Two studies were published in 2007, five studies in 2009, eight in 2010, four in 2011, and five in 2012.

### Question 1: What Percentage of Physicians Has Been Rated?

First, we investigated the number of ratings on physician-rating websites. One study estimated the number of ratings on physician-rating websites from a national perspective. Data for US physicians obtained from RateMDs showed that 16% (112,000 out of approx. 700,000) of national physicians were assessed by January 2010 [7]. Some studies regarded a sample of physicians to conduct analyses. Lagu and colleagues showed that 81 out of 300 Boston physicians had been rated (27%) [2]. In another study, Mostaghimi and colleagues counted that, out of 250 randomly selected internal medicine physicians, 53 physicians (21%) were rated on Healthgrades, 13 (5%) on RateMDs, and 1 physician on Wellness (0.4%), respectively. Most of the 250 physicians had still not been rated (Healthgrades: 69%, RateMDs: 61%, Wellness: 98%, respectively) [23]. In 2009, the percentage of rated physicians for ten different specialty/region combinations on five German physician-rating websites varied between 0% (eg, for urologists in Frankfurt) and 100% (radiologists in Hannover). The overall mean percentage of rated physicians was reported to be at between 3.36% (Patienten-empfehlen-Ärzte) and 25.78% (Medführer), respectively [21]. In a recently published study, between 3% and 28% of a random sample of physicians had been rated at least once [24].

### Question 2: What Is the Average Number of Ratings on Physician-Rating Websites?

Regarding the average number of ratings per physician on physician-rating websites, results for the American physician-rating website RateMDs were reported to be 2.7 mean ratings (range = 1-103) in 2009 [25] and 3.2 in January 2010 [7]. Nearly half of the physicians had only a single rating on RateMDs in 2010, and the number of physicians with five or more ratings was 12.5% [7]. For a sample of 300 Boston physicians, 190 reviews had been posted in total, ie, the mean number of ratings was 0.63 per physician. If only rated physicians (n=81) were analyzed, the mean number increased to 2.35 ratings [2]. Regarding a subsample of 250 randomly selected physicians in Boston, between one and four reviews could be found with 29% on Healthgrades, 39% on RateMDs, and 2% on Wellness, respectively. On Healthgrades, only 3 physicians (2%) had more than five reviews; no physician with five or more ratings could be found on RateMDs or Wellness [23]. German results from 2010 show similar findings; the number of ratings for physician-rating websites was reported to be 600,000 for Jameda, 450,000 for DocInsider, 150,000 for Arztauskunft, and 73,000 for Imedo, respectively. Compared to the total number of physicians in the German outpatient sector (approximately 150,000), the highest mean of approximately four evaluations per physician could be tracked on the website Jameda [6]. Another study determined a mean number of ratings of between 1.1 and 3.9; the maximum number of ratings per physician varied across the physician-rating websites at between 3 and 27 [24].

### Question 3: Are There Any Differences Among Rated Physicians Related to Socioeconomic Status?

There is little evidence available to answer the question of whether there are differences among rated physicians according to their socioeconomic status. Only three studies provided evidence relevant to this question. One previously published study showed that 74% of rated physicians were male (national average 72%). It was also shown that physicians who were board certified, and those who had at least one paid malpractice claim, were more likely to be rated. The authors could further show that younger physicians were much less likely to be rated. Graduates of more highly ranked medical schools and those of lower-ranked medical schools were rated with nearly the same frequency [7].

With respect to subgroups of physicians, Lagu and colleagues determined the number of rated generalists (37) and subspecialists (38) to be quite similar [2]. In absolute terms, primary care physicians were more likely to be rated than other specialties such as surgeons or obstetrics/gynecologist [7]. Another study showed that nonsurgical subspecialties, as well as OBGYN (Obstetrics Gynecology) & IVF (in-vitro fertilization), were most rated (22% and 19%, respectively). The lowest percentage of ratings was determined for doctors specialized in physical medicine and general surgery (1% and 2%, respectively) [25]. In relative terms, compared with the national physician composition, rated physicians were most likely to be obstetrician/gynecologists (32%). The likelihood of being rated for other specialties was calculated as follows: 25% of medical specialists, 20% of surgeons, 16% of primary care physicians, and 7% of physicians classified as other specialists (such as radiologists, pathologists, and anesthesiologists) [7]. Black et al showed that nonsurgical subspecialty and internal medicine physicians have been rated most (28% and 13%, respectively). In contrast, general surgery and physical medicine physicians have been rated the least (3% and 1%, respectively) [25]. Also, the numbers of individual ratings varied across specialty; the highest numbers were counted for OBGYN and IVF, dermatology, and cosmetic surgery (mean 4.4 individual ratings) and the lowest for pediatrics and general surgery (mean 1.8 ratings) [25].

### Question 4: Are Ratings More Likely to Be Positive or Negative?

In total, six studies provide information on the results of the ratings. Two studies focused on the US site RateMDs and found the overall reviews to be quite positive. On a scale of 1 to 5, the mean score was reported to be 3.93 [7] and 3.82 [25], respectively. A comprehensive analysis of German physician-rating websites confirmed that most ratings were positive. Here, the mean rating was between 1.1 and 1.5 (3-point scale, 1 “good”, 3 “poor”) [24]. When assessing the 10 most commonly visited US physician-rating websites, the aggregated mean ratings were as follows [22]: 77 out of 100 when using a 100-point scale (SD 11), 3.84 out of 5 (77%) for sites using a 5-point scale (SD 0.98), and 3.1 out of 4 (78%) for sites using a 4-point scale (SD 0.72). It was further reported that the percentage of reviews rated ≥75 on a 100-point scale was 61.5%, ≥4 on a 5-point scale was 57.74%, and ≥3 on a 4-point scale was 74.0% [22]. On RateMDs, 45.80% of the physicians received the best score and only 12% were rated with the worst score [7]. Other studies do not provide a mean rating but give further information about the percentage of positive and negative reviews. Lagu und colleagues did the same when they reported that the vast majority (88%) of reviews were positive, only six percent were negative, and six percent were neutral [2]. On Canadian RateMDs, 70% of the comments were reported to be favorable and about 30% comments were negative [26].

Exploring in more detail, the rating differences between physician groups was assessed in some studies. Gao et al found similar mean ratings for physicians in primary care (4.02), medical specialties (3.96), surgeon and surgical specialties (3.89), and obstetrician/gynecologists physicians (4.01). They further demonstrated that physicians listed within the group of other specialties had lower ratings (3.59) [7]. Others reported the highest mean scores for pediatricians, general surgery, and subspecialty surgery (4.22, 4.10, and 4.07, respectively) [25]. Lagu et al determined that generalists and subspecialists had a similar percentage of positive, negative, and neutral reviews [2]. Furthermore, male physicians, younger physicians, board-certified physicians, and those graduating from a top-50 medical school were shown to have statistically significant better ratings [7].

### Question 5: What Significance Do Patient Narratives Have?

Patients have the choice of writing narrative commentaries in free text form on 86% of English-language and German-language physician-rating websites [14]. Physicians’ critiques often concern these narratives, as they might provide the opportunity for doctor-bashing, defamation, etc. However, obtaining actionable information might help physicians to change communication style, facility, or staff. Such information may be better obtained by those narratives, rather than by a scaled survey displaying numbers or stars. A single quantitative rating of 1 out of 5 stars does not provide further assistance for improvement. But, if comments show that the exam rooms were dirty, then the provider will better understand the low rating [22].

Numbers on how many physicians have been rated by means of a patient narrative are quite scarce. According to US evidence, there is at least one narrative rating for approximately 17% of physicians [2]. Alemi and colleagues showed that the mean number of patient narratives per physician for a sample of 200 rated physicians by means of a patient narrative was 9 (range from 1 to 57, SD 8.10) [27]. Furthermore, the authors found that narratives were mostly positive (89%) [2]. In another study, Lopez et al qualitatively analyzed 712 narratives for internists and family practitioners from RateMDs and Yelp and found that 63% of the narratives contained positive comments [8]. In an analysis of 995 narratives from RateMDs, it could be determined that 69% (688) were praise, 21% (210) were complaints, and 10% (97) were both [27]. This result was confirmed by Black et al, showing that positive terms (54.1%) were more frequent than negative terms (16.0%) [25]. Thereby, the five most common positive terms were good, knowledgeable, best, excellent, and wonderful. In contrast, the most common negative terminology found was rude, bad, worst, horrible, and terrible [25]. The mean length of the narratives on RateMDs was 19.3 words [25]. Finally, Alemi and colleagues coded narratives with several reasons for dissatisfaction into nine categories, such as (1) physician-related concerns, (2) staff-related issues, (3) getting in to be seen, etc. As a result, most comments were related to aspects of category 1 (eg, doctor’s advice and treatment, time doctor takes, explanations provided by the doctor) and category 2 [27].

### Question 6: How Should Physicians Deal With Physician-Rating Websites?

Many physicians are uncertain about how to deal with physician-rating websites. Authors seem to agree that physician-rating websites will play a major role in health care in the future, and physicians should therefore not underestimate, but instead recognize, the popularity of such websites [23,25,28,29]. Physicians should be further aware of the fact that not only patients, but also insurance companies or even other physicians check these sites; the latter, for instance, to get information about job candidates [30].

Physicians should perform “self-audits” on popular physician-rating websites to search for available information [23,30], like their scores [29]. Therefore, it may be helpful if a staff member monitors these sites on a regular basis. If nothing else, physician-rating websites often provide incorrect demographic information (eg, incorrect address, links to old practices, opening hours), which should be corrected [23,30]. Next, physicians should use the ratings in order to evaluate their patients’ satisfaction [31]. Since existing measures of patient experiences do not seem to facilitate a good understanding for health care providers, personalized feedback on physician-rating websites may be advantageous. Rather than departmental reports or annual surveys, anonymous Internet-based reviews may help health care providers improve the quality of care [25]. Furthermore, measures such as medical training rarely give the opportunity to hear what patients want or value because in the real workplace, disappointed patients rarely tell doctors their true opinions. So, patients’ true thoughts on what makes a good doctor, what they value, etc., can be understood [29].

In the case of negative reviews, it is best not to respond online to try to refute the negative review point by point [30]. Further, negative reviews may help providers to create a more patient-centered office environment. Negative interpersonal reviews underscore the importance of a well-perceived bedside manner for a successful patient-physician interaction. Staff, access, etc., affect patient´s reviews as well [8]. However, the challenges of finding a remedy for negative ratings are daunting. An alternative option is to treat the problem before it becomes a problem. The legal company Medical Justice offered to provide doctors with a contract for treatment that includes a clause requiring patients to ask their doctor’s permission before posting a review to a website [30,32,33] (it is worth mentioning that the company has since stopped that practice). Another approach is to politely encourage satisfied patients to submit their own reviews on the most popular physician-rating websites [26,30]. Additionally, positive comments from patients should be posted on one’s own website [30]. Finally, physicians should not make a referral decision based upon results on physician-rating websites, as results related to patient satisfaction and outcome measures are not risk-adjusted and therefore cannot be regarded as reliable [6].

### Question 7: What Major Shortcomings Do Physician-Rating Websites Have?

Next, we discuss the major shortcomings of physician-rating websites.

1. Due to incomplete databases, it is shown that many physicians are not even listed on physician-rating websites [34,35]. For example, out of a random sample of 298 German physicians, between 75% and 98% of the physicians could be found [24].
2. On most physician-rating websites, only a small number of physicians have been rated so far [6,23,31,32,35]. As mentioned previously, only 16% of practicing US physicians have received at least one rating on RateMDs in 2010 [7], and only a low number of physicians has more than one rating (eg, only 2% had more than five reviews on Healthgrades in 2008) [23].
3. Patient opinions are unstructured, and ratings systems, as well as the presented information, are different on each physician-rating website [14,22,24]. One study showed that 35 different dimensions of care were rated on physician-rating websites [22]. Thus, meaningful information cannot be provided [31], and conducting physician-patient review meta-analysis or comparisons is difficult [24,35].
4. There is still no (gold) standard for surveys implemented on physician-rating websites for measuring patient satisfaction [14,24,28]. Some authors suggest that long surveys with preset questions are missing a great deal of information and force patients to distort their ideas to fit the questions asked [27]. In addition, star-rating systems may be crude and have dubious validity in the way that different categories are aggregated into an overall score [33]. One study showed that surveys vary significantly with respect to certain quality parameters in order to identify a good doctor’s practice [21].
5. Although a broad range of information is available on many physician-rating websites, the data are unlikely to reflect the quality of a physician. Most information is related to structural quality and patient satisfaction. Furthermore, significant measures such as outcomes and patient satisfaction are not risk-adjusted and, thus, are not likely to reflect the quality of care, but more the case mix of patients served [6].
6. Abuse is likely on physician-rating websites [6,21,28], and this leads to potential damage for both doctors (defamation) and patients (misinformation). As individuals can rate anonymously, it is impossible to tell if the rater is a patient or someone posing as a patient [29,32,33]. However, it is worth mentioning that physicians also seem to manipulate information on physician-rating websites [2].
7. Feedback, delivered anonymously*,* has limited ability to be related to specific incidents. So, it is unlikely that a doctor can learn from posted comments [36].
8. In case physicians disagree with a comment, they may not be able to respond to negative reviews*,* as they are bound by privacy laws and a duty to preserve the confidentiality of patient information [29,37]. In addition, only a few physician-rating websites allow physicians to respond to negative comments [37].
9. There is still a great lack of evidence of physician-rating websites’ effects on physicians’ performance, patient outcomes, or the public’s trust in health care [20]. There is further a Iack of knowledge on how physician-rating websites might be used by patients, why they are used, and the usefulness of the information gathered [38].
10. In general, the role of patients as reviewers of health care quality is still seen controversially: one argues that patients are not skilled or knowledgeable enough to assess the technical quality of care received [37,38]. Others state that patients’ experience is an important component of measuring the quality of care [38].

### Question 8: What Recommendations Can Be Made for Further Improvement of Physician-Rating Websites?

Several strategies have been suggested for further improvement of physician-rating websites. These address some of the limitations mentioned above:

1. Some authors discuss whether a simple One Feedback Question containing a single question such as “Would you recommend Dr X to a loved one?” may be as useful as the multitude of specific questions. The authors base their recommendation on the fact that there is a high correlation between the overall rating and the other dimensions of care rated [22]. Alemi et al suggest a 2-question survey: the “Minute Survey”. The first question asks patients to rate their overall experience. The second question asks: “Tell us what worked well and what needs improvement” [27].
2. Many surveys on the physician-rating websites should be revised to improve the usefulness of the ratings [21]. Here, certifications from professional societies and public institutions entailing not only formal and legal standards, but also specifications for a suitable representation and operationalization of patients’ experience and satisfaction were proposed. Therefore, a transparent process allowing participation by various stakeholders is essential [14].
3. Narrative comments to allow patients to write in specific feedback should be integrated in order to: (1) enable peer-to-peer communication amongst users [24], and (2) provide physicians with actionable information for change (see above) [22].
4. Patient narratives should be moderated, ie, there should be an option for the health care provider to comment on the rating [32,33,37]. Only then could a feedback loop be generated between patients and providers that would create value for both patients and providers [33,37].
5. Additional information should be considered on the physician-rating websites such as number of published scientific articles, outcome measures, clinical quality related to quality indicators, numbers treated with a certain disease, etc. [6,28]. Medical malpractice information should be addressed if the information source is recognized as authoritative (eg, licensing boards) [32]. Outcome measure scores must be risk-adjusted [6].
6. A minimum number of ratings (eg, 5-10) should be determined before publication is carried out [6,20,37]. This would reduce the impact of extreme opinions, and peer review would allow for the differentiation and elimination of defamations [20].
7. Certain quality strategies should be established to advance measures against fraud [21], eg, to remove ratings when meeting certain conditions—an IP address is traced to a medical practice, a lot of postings appear to come from the same source [33], or to apply adequate word filters and manual provider review before publication [21].
8. Quality standards for physician-rating websites should be considered by the providers of the websites. An example of this would be the quality criteria list developed by the German Agency for Quality in Medicine (ÄZQ), containing 40 questions and defining main quality standards regarding data privacy, transparency in terms of operators and funding, a clear and understandable assessment procedure, etc. [31].
9. physician-rating websites should be specifically tailored to the needs of vulnerable subgroups of the population. Preferably, aspects such as accessibility and the clarity of information should be improved [20].
10. Rules of behavior should be stated on each physician-rating website [37]. One example can be found on NHS Choices (eg, Category 12 Conduct, b. Postings should relate to your own personal experience).

Further recommendations advise that inappropriate content must be edited, users must register with an email [37], physician-rating websites should not contain advertising or official messages, as consumers value independence [38], and that ratings must be transmitted to the provider, albeit anonymously [38].

## Discussion

Physician-rating websites have been gaining much attention in many industrialized countries recently [7]. Discussions about prevalence of these websites, current usage, the main shortcomings, whether physicians have to worry about these rankings, and how physicians should handle these websites have frequently been raised. The aim of this review was thus to provide an overview of the empirical evidence and expert opinions, which were published in peer-reviewed journals. This paper adds to the literature by summarizing published knowledge with respect to eight ex-ante defined questions, which are deemed important in this context. To our knowledge, this is the first detailed systematic review related to physician-rating websites.

### Question 1: What Percentage of Physicians Has Been Rated?

Five papers were identified, and they all concluded that only a small percentage of physicians have been rated so far on a physician-rating website (eg, 16% of US physicians on RateMDs). As a result, the ratings shown are not likely to be representative of average patient experiences or consumers [6,23,29,30,36,37]. However, physician-rating websites have been gaining an increasing number of ratings over the last years (a 100-fold increase in the United States from 2005 to 2010) [7]. One reason for the low usage might be that patients are still unaware of these websites. A representative survey of 2048 German citizens showed that only 10% of respondents had used physician-rating websites in 2011; however, the number in 2010 was only 7% [39].

### Question 2: What Is the Average Number of Ratings on Physician-Rating Websites?

Regarding the mean number of ratings on physician-rating websites, US results were reported to range between 0.63 [2] and 3.2 ratings per physician [7]. German results ranged between 0.5 and 4 ratings per physician [6,24]. Thereby, most ratings are given for a low percentage of physicians, meaning that most physicians still remain unrated and those rated have a larger number of ratings. A large US study reported that half of the physicians had only a single rating, and the number of physicians with five or more ratings was 12.5% on RateMDs in 2010 [7]. Consequently, the benefit of such sites for patients still remains limited because more physicians must be rated. The mean number of ratings has to increase to provide a larger benefit to society. However, this might be solved with an increasing awareness level of rating portals.

### Question 3: Are There Any Differences Among Rated Physicians Related to Socioeconomic Status?

In total, three studies provided evidence on this question. Certain factors seem to increase the likelihood of being rated on a physician-rating website, such as being older, being male, being board certified, and having at least one paid malpractice claim. Furthermore, some specialties such as primary care physicians and obstetrician/gynecologists seem to influence the likelihood of being rated [7]. Specifically, this includes physicians who have more direct patient contact or those who treat population groups who are more likely to use the Internet actively, such as a younger and female patient population [7].

### Question 4: Are Ratings More Likely to Be Positive or Negative?

Some authors expressed concerns whether physician-rating websites might become a channel for disgruntled patients [7]. However, this cannot be confirmed, since international results showed that most ratings express a positive opinion about physicians. One US study determined 88% positive, 6% negative, and 6% neutral ratings [2]. A comprehensive US study confirmed this by showing the mean ratings according to different scoring scales [22]. In sum, studies confirmed that most reviews are on the extreme end, meaning either positive or negative. The studies suggest that most ratings are positive and therefore that some physicians’ concerns may be exaggerated [37].

### Question 5: What Significance Do Patient Narratives Have?

Our results show that, up to this point, a low number of physicians have been rated by means of a patient narrative; one US study reported a number of 17% [2]. Furthermore, most opinions in narratives are positive (numbers range between 63% and 89%, respectively) [2,8,27]. Physicians’ concerns are about “doctor-bashing”, defamation, etc. However, no evidence has been found to sustain this concern. Adequate measures seem to be in place before comments are published. German physician-rating websites were reported to have implemented adequate word filters, manual provider review, etc. [21], which seem to be effective. Thus, the risk of defamation of physicians in patient narratives seems to be low. Furthermore, it is worth mentioning that physicians also seem to manipulate information on physician-rating websites [2].

### Question 6: How Should Physician Deal With Physician-Rating Websites?

According to the literature, physician-rating websites might play a major role in future health care; therefore, physicians should not underestimate, but instead recognize, the popularity of such websites [23,25,28,29]. We showed that getting an overview of the physician-rating websites is recommended, as well as staying on top of the available information on a regular basis. However, no general guidelines are available on how to deal with physician-rating websites. While some may continue to ignore physician-rating websites (due to higher age, little engagement, etc.), others may seek this information in order to be informed, and still others will try to obtain as many positive reviews as possible.

### Question 7: What Major Shortcomings Do Physician-Rating Websites Have?

While some flaws are of minor importance, there are also some very major ones. In our estimation, the most important flaw is that physician-rating websites are not able to identify the best physician for a specific intervention or disease. Therefore, the information provided is both too little and not (disease) specific enough. However, it should be debated whether physician-rating websites are really supposed to achieve that. It is more likely that physician-rating websites can give some limited impression of, and only of, patient satisfaction and some structural information. But even these results have to be viewed with caution [6,21,28].

### Question 8: What Recommendations Can Be Made for Further Improvement of Physician-Rating Websites?

Frequently discussed improvement recommendations relate to the feedback survey. Some argue that a long and detailed survey is necessary to assess the quality of care received. However, the more questions a patient has to answer, the less likely they are to complete the survey [22]. Therefore, a single question is supposed to be sufficient. However, internationally established and validated instruments, such as the Patient Satisfaction Questionnaire from RAND Health (50 items), are more detailed and contain more questions to derive specific results. Consequently, from a researcher’s point of view, the application of validated instruments should be preferred.

It also seems to be the predominant opinion that narrative comments should be integrated on physician-rating websites [22,24]. Of course, this also means that the physician-rating website provider has to establish certain quality measures. However, from the point of view of a patient or physician, the benefits justify it. If physician-rating websites are intended to provide real support to patients, then additional information has to be integrated on the websites, and outcomes must be risk-adjusted [6]. For other sectors of health care (eg, hospitals), risk-adjusted outcome measures are increasingly available. In Germany, the Aqua Institut (www.sqg.de) provides quality indicator data about most German hospitals, and a growing segment of this data is available for PR. In the United States, the Centers for Medicare and Medicaid Services (CMS) offer the website, Hospital Compare. Thus, in the outpatient sector, both outcome information and ratings from patients are available. However, measures about physicians are less available. In Germany, the Aqua Institut has recently started to collect outcome measures about physicians. In the United States, the CMS recently launched Physician Compare, a website publishing data on quality measures for covered professional services provided to Medicare beneficiaries. Consequently, we expect a growing number of risk-adjusted outcome quality indicators to be available in the future for PR about physicians’ quality as well.

### Limitations

Our systematic review has several limitations. It was based on searches in seven databases, and we included articles containing at least an abstract in English. So, it is possible that additional papers exist that were not included. We further concentrated on papers dealing with websites on which individual physicians can be rated. Consequently, knowledge coming from the assessment of websites on which provider organizations or entire hospitals can be rated is not included in our review. Due to the time constraints of our research (up to May 2012), it may be the case that some recently published papers are not included. By focusing only on peer-reviewed literature, we may have missed information in the grey literature that could also have been of interest in attempting to answer some of our questions. Furthermore, due to study heterogeneity, we did not carry out any study appraisal. Before conducting this review, we conducted some interviews with physicians, patients, and physician-rating website providers to get an impression of important questions. However, there may be other relevant questions to discuss, which we did not identify.

### Conclusions

To our knowledge, this is the first systematic review of physician-rating websites. Our research shows that the current usage of physician-rating websites, with respect to the number of ratings, is still low but is increasing. Most ratings express positive opinions; this is true for the results of both predetermined rating systems and patient narrative comments. Although negative ratings were mentioned across the different studies, there was no evidence that they are worse via this particular mechanism. Consequently, we could not find any evidence of doctor-bashing in any of the studies. Physicians should not ignore these websites but instead should monitor the available information and use it for internal and external purposes. Several shortcomings limit the significance of the results published on physician-rating websites, and some suggestions on improvement were shown to address them.

The literature suggests several fields for future research. These include investigating how to generate and communicate adequate information for patients. Although a lot of information is provided on physician-rating websites, it remains unclear why patients use these platforms and what the relevant information is about physicians that should be considered for publication on physician-rating websites [28,38]. In this regard, it remains unclear whether patients actually understand the information provided enough to make correct choices [37,38]. Furthermore, research should specifically consider the requirements of disadvantaged people (eg, culture, sex, age, education, socioeconomic group, disability, and health status) to find out whether there are any barriers for certain population groups when seeking and using information provided on physician-rating websites [20,28,38]. The cost-effectiveness of physician-rating websites must be investigated to assess whether [28] the effects of the websites (eg, patient steerage, quality improvement) are large enough to be viewed as money well spent. In this context, the usability of physician-rating websites seems to be crucial. Studies have shown these sites to be neither user-friendly nor patient-centered [2]. Others state that the handling of some physician-rating websites is too complex for some users with respect to the clarity of the physician-rating website or offered search options [34]. Finally, policy makers could contribute to the development of such sites by establishing a regulatory framework to foster the availability of data assessing the quality of care of physicians. This data then could be used for PR. Therefore, experience from the German inpatient sector (see above) could be used.
